# *Matricaria chamomilla* Essential Oils: Repellency and Toxicity against Imported Fire Ants (Hymenoptera: Formicidae)

**DOI:** 10.3390/molecules28145584

**Published:** 2023-07-22

**Authors:** Farhan Mahmood Shah, Dileep Kumar Guddeti, Pradeep Paudel, Jian Chen, Xing-Cong Li, Ikhlas A. Khan, Abbas Ali

**Affiliations:** 1 National Center for Natural Products Research, The University of Mississippi, University, MS 38677, USA; fshah@olemiss.edu (F.M.S.); gdileepkumar19@gmail.com (D.K.G.); ppradeep@olemiss.edu (P.P.); xcli7@olemiss.edu (X.-C.L.); ikhan@olemiss.edu (I.A.K.); 2 Biological Control of Pests Research Unit, USDA-ARS, Stoneville, MS 38776, USA; jian.chen@usda.gov

**Keywords:** chamomiles, essential oils, α-bisabolol, invasive ants, repellents, toxicants

## Abstract

*Matricaria chamomilla* flower essential oils (EOs) blue Egyptian (EO-**1**), chamomile German CO2 (EO-**2**), and chamomile German (EO-**3**) and the pure compound α-bisabolol were evaluated against red imported fire ants (RIFA), *Solenopsis invicta* Buren, black imported fire ants, *S. richteri* Forel (BIFA), and hybrid imported fire ants (HIFA) for their repellency and toxicity. A series of serial dilutions were tested starting from 125 µg/g until the failure of the treatment. Based on the amount of sand removed, EO-**1** showed significant repellency at dosages of 7.8, 7.8, and 31.25 µg/g against RIFA, BIFA, and HIFA, respectively. EO-**3** was repellent at 3.9, 7.8, and 31.25 µg/g against BIFA, RIFA, and HIFA, whereas α-bisabolol was active at 7.8, 7.8, and 31.25 µg/g against BIFA, HIFA, and RIFA, respectively. DEET (N, N-diethyl-meta-toluamide) was active at 31.25 µg/g. Toxicity of EOs and α-bisabolol was mild to moderate. For EO-**1,** LC_50_ values were 93.6 and 188.11 µg/g against RIFA and BIFA; 98.11 and 138.4 µg/g for EO-**2**; and 142.92 and 202.49 µg/g for EO-**3**, respectively. The LC_50_ of α-bisabolol was 159.23 µg/g against RIFA. In conclusion, *M. chamomilla* EOs and α-bisabolol offer great potential to be developed as imported fire ant repellents.

## 1. Introduction

Red imported fire ants (RIFA; *Solenopsis invicta* Buren), black imported fire ants (BIFA; *S. richteri* Forel) (Hymenoptera: Formicidae), and hybrid imported fire ants are notorious pests, impacting humans, wildlife, pets, and livestock through their venomous stings [1,2]. Imported fire ants damage planted field crops [3,4,5] and deteriorate circuitry and electrical poles/sign boards because of mound buildings [6]. Their increasing infestations have shown negative ecological impacts, including predation and competition, impacting the local biodiversity and disturbing ecological processes [7,8]. Imported fire ants invaded the United States from their native South America in the early 1900s [9]. Currently, RIFA are present in 13 states and Puerto Rico [10], whereas BIFA are present along the northern boundary of RIFA’s distribution range [11,12]. Extensive hybridization occurs between RIFA and BIFA along their population boundaries, resulting in the development of HIFA in the Southern States [11,12,13]. Because of their presence in China, Australia, and many other regions of the world, RIFA are considered to be one of the most serious invaders that has spread through global trade and transportation. In the United States, the total cost associated with imported fire ants is estimated at USD 6 billion, annually [14]. The United States has a federal quarantine law in effect to limit fire ants spread to non-infested areas (http://www.aphis.usda.gov/ppq/ispm/fireants/index.html), accessed on 29 October 2022.

Currently, the common methods used for control of imported fire ants include baiting [15] or treating individual mounds with contact synthetic pesticides [16]. Although these methods offer potential to control fire ants, repeated, long-term use of these chemicals tends to create health and safety concerns. It can affect non-target organisms and pollute the environment. Imported fire ants have the potential to develop detoxification mechanisms that can promote pesticide resistance [17]. Particle-covering behavior that complicates the contact toxicity of synthetic pesticides has also been reported in fire ants [18]. Post-treatment mound relocations warrant multiple applications [15]. Based on these complications, there is a need to develop alternative insect management tools [15,17,18,19,20].

Plant-based natural products as alternatives to synthetic insecticides have been the major focus of current research. Plant-derived bio-active compounds are renewable, cheap, rapidly biodegradable, target specific, and environmentally friendly and could be used as effective alternatives against pests of great medical and veterinary importance as well as agriculture [21,22,23,24]. These compounds can act as attractants, antifeedants, repellents, oviposition modifiers or indirectly affect insects by changing key metabolic processes that leads to their rapid death [25,26,27,28,29,30]. In fire ants, natural repellents can be very useful because of their ability to inhibit the digging behavior that can potentially be used as an effective tool in quarantine treatments in sensitive areas, in and around homes, hospitals, laboratory stock, circuitry, and storage conditions [1,6,31] where use of conventional insecticides is considered unsafe. For their high target specificity, low quantity usage, and affinity characteristics of the active compounds, natural products are relatively non-toxic as compared to synthetic chemicals [32] and safe to use in preventing the spread of imported fire ants [33].

Many plant materials have been screened for potential use as natural repellents against imported fire ants. Fire ant digging/nesting behavior has been focused on for the assessment of repellency [34]. Drenching mound soils with repellent d-limonene from citrus oil inhibited ant activities [35]. Imported fire ants leave the mounds that were treated with repellent mint oil granules [36]. Worker ants removed significantly less sand when exposed to the sand treated with *Magnolia grandiflora* essential oil and its pure compounds, e.g., 1-decanol and 1-octanol [37]. Treating flowerpots with methyl isoeugenol prevented ant nesting for almost over a month [38].

German chamomile, *Matricaria chamomilla* L., native to Southern and Eastern Europe, is a herb in the family Asteraceae that is famous for its medicinal values [39]. Several compounds from *M. chamomilla* have been identified to show anti-insect activities [40,41]. In our natural product screening program, *Matricaria chamomilla* essential oils of various chemotypes and their major compounds showed toxicity and repellency against mosquitoes [40,41]. Based on repellent data against mosquitoes, different chamomiles essential oils were tested for their repellency and toxicity against imported fire ants. Out of many essential oil samples that were tested in our screening program, three essential oils showed repellent activity and were selected for further testing. This manuscript presents data on toxicity and repellency of three *M. chamomilla* flower essential oils and α-bisabolol against RIFA, BIFA, and HIFA workers.

## 2. Results

### 2.1. Chemical Composition

**Figure 1:**
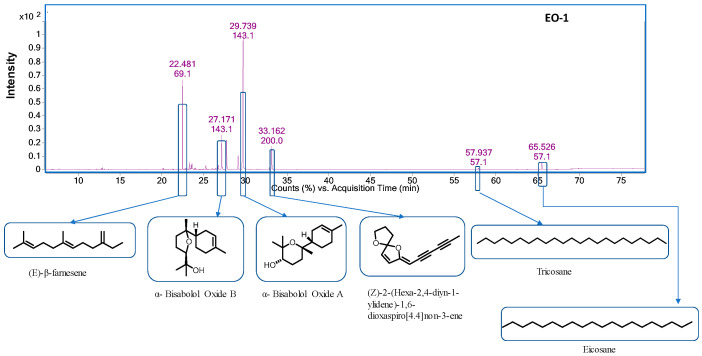
GC-MS chromatogram of chamomile EO-**1**.

**Figure 2:**
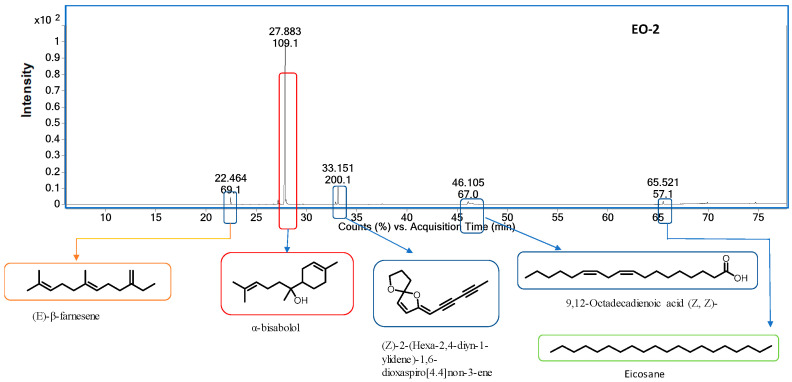
GC-MS chromatogram of chamomile EO-**2**.

**Figure 3:**
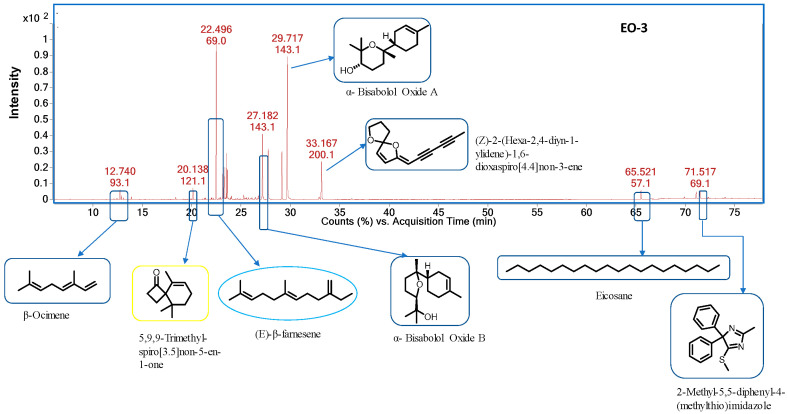
GC-MS chromatogram of chamomile EO-**3**.

Chemical compositions of the three chamomiles EOs are given in Table 1, while their GC-MS chromatograms are shown in Figure 1, Figure 2 and Figure 3. GC-MS analysis identified 30 compounds. We studied the retention indices (RI) of the components experimentally by using a homologous series of n-alkanes from C_8_–C_20_ and C_21_–C_40_ standards. Oil constituent’s identification involved comparisons of retention indices (RI_Exp._) with those already available in the literature (RI_Lit._) and their mass spectra matched with the NIST2014 library. The percentage composition of a particular component in each EO was assessed using automatically integrated peak areas of the GC-FID signal.

EO-**2** contained α-bisabolol (81.85%), (Z)-2-(hexa-2,4-diyn-1-ylidene)-1,6-dioxaspiro [4,4] non-3-ene (6.98%), 7-methoxycoumarin (2.60%), α- bisabolol oxide B (1.45%), (E)-β-farnesene (1.39%), and 9,12-octadecadienoic acid (Z, Z)-(1.39%) as major compounds. EO-**1** and EO-**3** had similar major metabolites except that bisabolol oxide A was 49.19% and 27.83%, in EO-**1** and EO-**3**, respectively.

### 2.2. Digging Bioassay

Mean weight (g) of sand removed by the workers in a digging bioassay treated with different concentrations of *M. chamomilla* EOs, its selected pure compound, and the positive control DEET is presented are Table 2. Based on the amount of sand removed, EO-**1** showed significantly higher repellency than ethanol at dosages of 125–7.8 µg/g against RIFA and BIFA, whereas the activity at 62.5–31.25 µg/g was similar to ethanol against HIFA. EO-**2** showed significantly higher repellency than ethanol at dosages of 125–15.6 µg/g against RIFA, whereas the activity at 15.6–3.9 µg/g was similar to ethanol against BIFA and HIFA. EO-**3** showed significantly higher repellency than ethanol at dosages of 125–3.9 µg/g against BIFA, whereas the activity was similar to ethanol at 3.9 µg/g against RIFA and at 31.25 µg/g against HIFA. α-Bisabolol showed significantly higher repellency than ethanol at dosages of 125–7.8 µg/g against BIFA and HIFA, whereas the activity was similar to ethanol at 15.6–3.9 µg/g against RIFA. Against BIFA and RIFA, DEET treatments showed significantly higher repellency than ethanol at dosages of 125–62.5 µg/g, whereas the repellency at 31.25 µg/g was similar with ethanol. In HIFA, the repellency of DEET was significantly higher at dosages of 125–62.5 µg/g, whereas the repellency at 31.25 µg/g was similar to ethanol.

### 2.3. Toxicity Bioassay

Toxicity data of *M. chamomilla* EOs, its pure compound, and bifenthrin at 24 h post-treatment against RIFA, BIFA, and HIFA workers are given in Table 3. EO-**1** showed toxicity with LC_50_ values of 93.60 and 188.11 µg/g against RIFA and BIFA workers, respectively. LC_50_ values of EO-**2** were 98.11 and 138.40 µg/g against RIFA and BIFA, respectively. EO-**3** showed LC_50_ of 142.92 and 202.49 µg/g against RIFA and BIFA workers, respectively, whereas LC_50_ value of α-bisabolol was 159.23 µg/g against RIFA workers. α-Bisabolol gave 30% mortality against BIFA at the highest screening dose of 250 µg/g. EO-**1,** EO-**2,** EO-**3,** and α-bisabolol gave 73%, 20%, 40%, and 80% mortality at the highest screening dose of 250 µg/g against HIFA. Bifenthrin with LC_50_ values of 0.03, 0.032, and 0.018 µg/g against RIFA, BIFA, and HIFA workers, respectively, at 24 h post treatment was more toxic than the essential oils. Based on LC_50_ values, toxicity of *M. chamomilla* EOs and α-bisabolol was significantly higher in RIFA as compared to BIFA.

## 3. Discussion

The chemical composition of chamomile EOs is complex and varies with differences in genetics, geographical distribution, harvest season, and the methods used for extraction [42,43]. α-Bisabolol and its oxides (α-bisabolol oxide-A and -B) are generally their major constituents and, based on the contents of these three compounds, chamomile EOs can be classified as chemotype A (bisabolol oxide A dominant), chemotype B (bisabolol oxide B dominant), chemotype C (α-bisabolol dominant), and chemotype D (α-bisabolol and bisabolol oxide A and B present in 1:1 ratio approx.) [39]. Extraction methods may also affect the chemical compositions of EOs. For example, the chamomile EOs when obtained by steam distillation, can be colored intensive blue (as observed for EO-**1** and EO-**3**) due to the presence of chamazulene, which originates from pro-azulenes (matricin and matricarin) at the increased temperatures and in the presence of organic acids contained in the flowers [44]. Likewise, extracts or EOs prepared from chamomile using supercritical carbon dioxide (CO_2_) extraction can be yellow (as observed for EO-**2**) because they lack chamazulene [44].

Numerous biological activities of chamomile EOs have been reported, and each biological activity is closely associated with a specific active constituent(s) [39]. Antioxidant and anti-inflammatory activities of chamomile EOs depend on the amount of α-bisabolol and its oxides [45]. The antioxidant effect of α-bisabolol oxides is stronger than α-bisabolol [46]. However, the anti-inflammatory activity of α-bisabolol is better than its oxides. In the current study, EO-**1** and EO-**3** belong to chemotype A due to the dominance of bisabolol oxide A, while EO-**2** is a characteristic chemical type C with an extremely high content (81.85%) of α-bisabolol. Analysis of the repellent activities and chemical compositions of the three chamomiles EOs indicated that α-bisabolol may be the primary active constituent responsible for the repellent activity observed for EO-**2**, which was confirmed by subsequent evaluation of α-bisabolol showing better repellency than EO-**2**. For the current study, we did not select bisabolol oxide A because this compound failed to show any significant repellency against mosquitoes in our natural product screening program [47].

Chamomile oils have been reported to show repellency against different species of insect pests. Höferl et al. [40] reported biting deterrent and repellent activities of the EOs from *M. chamomilla* against female *Aedes aegypti.* Aqueous and methanolic extracts of *M. chamomilla* were strong repellents against *Tribolium castaneum* (Herbst) (Coleoptera: Tenebrionidae) in stored wheat [48]. Al-Jabr [49] reported that *M. chamomilla* essential oil at 1% concentration in acetone repelled 81.94% of the *Oryzaephilus surinamensis* (L.) (Coleoptera: Silvanidae) for 48 h, and this essential oil at a concentration of 15% repelled 67% of *T. castaneum* adults for 180 min [50]. Cineole and d-camphor isolated from sweet wormwood (*Artemisia annua* L.) at concentrations of 100, 10, and 1 mg/kg were significant repellents against RIFA [51]. Camphor essential oil from *Cinnamonum camphora* Siebold were repellents to RIFA [52]. Hashimoto et al. [53] proved that microencapsulated allyl isothiocyanate could be used as repellents against RIFA. He et al. [38] reported that methyl isoeugenol could be used as a promising repellent against RIFA because treating flowerpot sand with methyl isoeugenol prevents the workers from nesting these flowerpots for over a month. The current study demonstrated repellency of *M. chamomilla* EOs against RIFA, BIFA, and HIFA. Higher repellency of these natural products as compared to DEET in the present study corroborates the findings of Ali et al. [37] who reported higher repellency of *Magnolia grandiflora* seed EO, and its pure compounds as compared to DEET in HIFA. There were differences among the repellency against RIFA, BIFA, and HIFA workers, which demonstrated that the repellency of various natural products could vary among imported fire ant species. These findings corroborate the findings of Chen et al. [54] who reported that repellency of callicarpenal and intermedeol varied among fire ant species. Our findings suggest that EO-**3** is the most active repellent against BIFA, and EO-**1** and α-bisabolol are promising against RIFA, whereas α-bisabolol was active against HIFA. This study is the first detailed report on the repellency of *M. chamomilla* EOs and α-bisabolol against imported fire ants.

Insecticidal activity of *Matricaria chamomilla* EOs has been reported against many species of insect pests. The EOs of *M. chamomilla* at a concentration of 0.75% (*w/w*) showed 100% mortality against *Oryzaephilus surinamensis* L. (Coleoptera: Silvanidae) when exposed to the treated wheat for 2 weeks [49]. Wheat grain treated with aqueous and methanolic extracts of *M. chamomilla* at a dosage of 1000 ppm/kg of grain caused 57% mortality in adult *T. castaneum* after 7 days of exposure [48]. EOs of *Matricaria recutita* L., with santolina alcohol (40.7%) and germacrene D (8.9%) as major contents, showed insecticidal activity against *Callosobruchus maculatus* (Fab.) [Coleoptera: Chrysomelidae], and at the dose of 1 μL/1 L of air, *M. recutita* essential oil induced a 70.7% and 60.1% reduction in oviposition and emergence [55]. EOs from *M. chamomilla* at a concentration of 15% caused significant mortality (LC_50_ = 7.78%) in adults *T. castaneum* at 24 h post-treatment [50]. Toxicity of EOs and α-bisabolol differed against three imported fire ant species. Based on LC_50_ values, EO-**1** and EO-**3**, and α-bisabolol, were more toxic to RIFA as compared to BIFA. The dose–response curves could not be developed for HIFA because all the samples showed less than 100% mortality at the highest screening dose of 250 µg/g and abruptly dropped to 0 % at the next serial dose at 24 h post-treatment. Still, the percent mortality data from this study can be used to predict the toxicity potential of EOs and the pure compound from *M. chamomilla* against HIFA workers. This is the first detailed report of the toxicity of *M. chamomilla* EOs and its pure compound against imported fire ant workers.

## 4. Materials and Methods

### 4.1. Chemicals and GC-MS

Chamomile oil Blue Egyptian (EO-1) was purchased from Perfumer Supply House (Danbury, CT, USA) and chamomile German CO_2_ essential oil (EO-2) and chamomile German essential oil (EO-3) were purchased from Eden’s Garden (Blaine, MN, USA). α-Bisabolol was purchased from Sigma Aldrich (St. Louis, MO, USA). GC-MS analysis of three EOs was performed in Agilent 7890 B GC system, which was equipped with a 5977A quadrupole mass spectrometer and with a 7693 autosampler (Agilent Technologies, Santa Clara, CA, USA). Samples were prepared at 10 mg/mL concentration in GC-MS-grade methylene chloride (Sigma-Aldrich, St. Louis, MO). The carrier gas Helium at a constant flow rate of 1.5 mL/min was used. The inlet temperature in split injection mode was set to 280 °C, with a 30:1 split ratio. The initial oven temperature program was set at 60 °C for 2 min, and was increased to 280 °C at a rate of 6 °C/min. Isothermal was at 280 °C for 10 min. The overall run time was 77 min. Agilent MassHunter software (version B.07.06) was used to perform data acquisition.

### 4.2. Ants

RIFA, BIFA, and HIFA workers were used in these bioassays. BIFA colonies were brought from Tunica County, MS-713, Hernando, MS 38632 (34°49′56.5″ N 90°12′55.6″ W). RIFA collection was made from Washington County, MS 38748 (33°9′31.2″ N 90°54′56.4″ W). HIFA workers were used from the mounds located under natural field conditions at University Field Station (University of Mississippi, 15 County Road 2078, Abbeville, MS 38601). Ant collection was brought to the laboratory and kept in plastic trays, whose top inner walls were coated with Insect a Slip (BioQuip Products 2321 Gladwick Street Rancho Dominguez, Compton, CA, USA) to prevent the escape of ants. Crickets and 25% honey-water solution were available as food to the ants. A test tube filled with water and plugged with cotton served as a water source. Moist sand-filled 45 mL fluted aluminum dishes (Fisher Scientific, 300 Industry Drive, Pittsburgh, PA, USA) were kept inside trays to serve as a digging substrate. The ants were maintained under laboratory conditions of 32 ± 2 °C temperature and 50 ± 10% relative humidity for one month before starting the bioassays. The ant species were identified based on venom alkaloid and hydrocarbon indices of the collected individuals [37,56].

### 4.3. Digging Bioassay

According to Deletre et al. [57], repellent refers to a chemical that causes an organism to make oriented movement away from its source, while in fire ants repellency refers to the suppression of digging behavior [34]. Because the inhibition of digging ability is the result of the contact, the ant workers fail to dig through, remove, or nest the soil/sand treated with effective repellents. This study used digging behavior of fire ants as a criterion for assessing repellency [37], assuming that a worker’s digging ability will depend on the repellent activity of the treatments. We followed the digging bioassay described by Chen [34] and modified by Ali et al. [37]. The tested arena consisted of a 150 mm × 15 mm petri dish, which contained four 2-mL Nylgene Cryoware Cryogenic vials glued at equal spaces on the bottom of the petri dish. To prevent the escape of ants, the inner walls of the arena were coated with Insect a Slip. Sand (Premium Play Sand, Plassein International, Longview, TX, USA) of the uniform size (500 microns) was used as a digging substrate. To remove any prior contamination, the sand was washed with de-ionized water and oven-dried at 190 ˚C for 6-h. Four g of sand was weighed in fluted aluminum (45 mL size) dishes (Fisher Scientific, 300 Industry Drive Pittsburgh, PA 15275). Treatments were applied in a volume of 100 µL/g of sand. Ethanol 100% was used as the solvent to prepare stocks and dilutions. Treatments were thoroughly mixed with sand. Sand in the control treatment received ethanol only. Once the solvent had evaporated completely, sand in each treatment was moistened by adding a 0.6 µL/g of de-ionized water and filled in the vials by using small spatulas. Treated sand vials were thoroughly packed to eliminate any spaces in the vials. Sand-filled vials were screwed to the bottom of the arena. On a dry weight basis, each vial contained 3.6 g of sand. Using a soft camel hairbrush, fifty ant workers were released per arena. All experiments were performed under laboratory settings of 25 ± 2 °C temperature and 50 ± 10% relative humidity. At 24 h post-treatment, sand from the arena vials was collected back into aluminum dishes, oven dried at 190 ˚C for 1 h, and weighed. EOs of *M. chamomilla,* a pure compound, and a positive control DEET (N, N-diethyl-meta-toluamide) were tested. A series of dilutions were tested starting from 125 µg/g until the failure of the treatment. Overall, three sets of the replicates were run on 3 different days. Sand removal data were analyzed by Analysis of Variance (ANOVA) (SAS 9.4, 2012) and means were separated by using Ryan–Einot–Gabriel–Welsch multiple range test (*p* ≤ 0.05; SAS 9.4 (2012)).

### 4.4. Toxicity Bioassay

The toxicity bioassay described by Ali et al. [37] was used for assessing mortality in imported fire ants. Briefly, this bioassay uses ant digging behavior to promote contact between ants and treated sand, leading to mortality at lethal dosages. Both the stock and dilutions were prepared in 100% ethanol. Three g of sand was weighed in fluted aluminum dishes (42 mL) and treatments were applied in a volume of 100 µL/g of sand. Sand in the control was treated with ethanol only. Once the solvent had evaporated, 0.6 µL/g of de-ionized water was added to moisten the sand. The moistened sand was then transferred into 60 × 15 mm Stackable Petri Dishes (KORD-VALMARK, Mfg by Bioplast Manufacturing, LLC. 128 Wharton Road, Bristol, PA, USA), whose inner walls were coated with Insect a Slip (BioQuip Products 2321 Gladwick Street Rancho Dominguez, CA 90220, USA). Ten workers were carefully released in each treatment. Continuous supply of moisture was ensured in each petri dish by adding a water-soaked cotton swab tip at 1 h post-treatment. The number of dead workers was recorded at 24 h post-treatment. LC_50_ values were calculated by using probit analysis (SAS 2012).

## 5. Conclusions

In conclusion, this is the first report on the insecticidal and repellent activities of essential oils of *M. chamomilla* against red, black, and hybrid imported fire ants. Removal of treated sand in digging bioassays, which may directly be proportional to inhibition of digging ability of the fire ants, was used as a criterion for repellency. Based on the above criterion, essential oils and α-bisabolol showed the potential to be significant repellents against the imported fire ants. All the natural products tested in this study, especially EO-**1**, EO-**3**, and α-bisabolol showed repellency higher than DEET. Further studies will be conducted to explore repellent/toxicant potential of these essential oils and the pure compound by testing in different formulations against imported fire ants in elaborate laboratory and field trials.

## Figures and Tables

**Table 1:** Chemical composition of three chamomile essential oils.

S. No	RT ^a^	RI ^b^ (Exp.)	RI ^c^ (Lit.)	Compound Name	Content%			ID ^d^
					EO-1	EO-2	EO-3	
1	12.74	1039	1044	β-Ocimene	−	−	0.80	RI, MS
2	12.89	1045		1,5-Heptadien-4-one, 3,3,6-trimethyl-	−	−	0.33	RI, MS
3	12.89	1045	1056	Artemisia ketone	0.30	−	−	RI, MS
4	13.88	1083	1086	α-Terpinolene	−	−	0.26	RI, MS
5	20.13	1342		5,9,9-Trimethyl-spiro [3,5] non-5-en-1-one	−	−	0.99	RI, MS
6	20.13	1342	1335	Elemene isomer	0.22	−	−	RI, MS
7	22.48	1450	1454	(*E*)-β-Farnesene	13.98	1.39	16.56	RI, MS
8	23.16	1481		3H-Pyrazol-3-one, 2,4-dihydro-2-methyl-5-phenyl-	−	−	2.98	RI, MS
9	23.28	1487	1430	(-)-β-Copaene	1.11	−	−	RI, MS
10	23.29	1488	1480	Germacrene D		−	2.93	RI, MS
11	23.51	1498	1505	α-Farnesene	0.67	−	4.06	RI, MS
12	23.61	1503		3,6-Dihydrochamazulene	1.15	−	−	RI, MS
13	23.62	1503	1500	Bicyclogermacrene		−	2.84	RI, MS
14	23.91	1516	1478	γ-Muurolene	0.25	−	−	RI, MS
15	24.05	1522	1522	Cadinene		−	0.53	RI, MS
16	25.27	1575	1577	Spathulenol	0.69	−	0.53	RI, MS
17	26.75	1633		1-epi-Bicyclosesquiphellandrene	0.86	−	−	RI, MS
18	26.76	1633		Bicyclo [4.4.0] dec-1-ene, 2-isopropyl-5-methyl-9-methylene	−	−	0.57	RI, MS
19	27.17	1648	1656	α- Bisabolol Oxide B	7.55	1.46	8.80	RI, MS
20	27.30	1653		Cyclofenchene	−	0.96		RI, MS
21	27.75	1670	1684	Bisabolone oxide A	7.38		7.13	RI, MS
22	27.88	1674	1685	α-Bisabolol	−	81.85	−	RI, MS
23	27.96	1677		7-Methoxycoumarin	−	2.60	−	RI, MS
24	29.13	1717	1730	Chamazulene	3.30	−	6.89	RI, MS
25	29.73	1735	1748	α-Bisabolol oxide A	49.19	−	27.83	RI, MS
26	32.92	1827		(*Z*)-2-(Hexa-2,4-diyn-1-ylidene)-1,6-dioxaspiro [4,4] non-3-ene	6.84	6.98	6.73	RI, MS
27	46.11	2106	2132	9,12-Octadecadienoic acid (*Z*,*Z*)-	−	1.39		RI, MS
28	65.52	2500	2000	Eicosane	0.71	1.02	1.35	RI, MS
29	71.12	2753		Methacrylic acid, 2,2,2-trichloroethyl ester	−	−	1.03	RI, MS
30	71.51	2771		2-Methyl-5,5-diphenyl-4-(methylthio) imidazole	−	−	1.26	RI, MS

^a^ Retention time in minutes; ^b^ experimental value for retention index; ^c^ literature values for retention index; ^d^ identification method; “−” represents trace amount or not detected.

**Table 2:** Mean weights (g) of treated sand removed by the workers of red imported hybrid fire ant, released in digging bioassays, with different concentrations of *Matricaria chamomilla* essential oils and a pure compound.

Conc. (µg/g)	Mean ± SE ^†^	*F*-Value	*p*-Value	Mean ± SE ^†^	*F*-Value	*p*-Value	Mean ± SE ^†^	*F*-Value	*p*-Value
	RIFA			BIFA			HIFA		
EO-1									
Control	1.66 ± 0.17 a	11.74	0.003	1.54 ± 0.37 a	8.36	0.008	-		
15.6	0.00 ± 0.00 bc			0.00 ± 0.00 c			-		
7.8	0.39 ± 0.16 b			0.50 ± 0.27 bc			-		
3.9	0.94 ± 0.35 ab			0.92 ± 0.19 ab			-		
Control	1.05 ± 0.04 a	86.18	<0.001	2.02 ± 0.14 a	180.09	<0.001	2.33 ± 0.21 a	4.15	0.023
125	0.00 ± 0.00 b			0.00 ± 0.00 b			1.01 ± 0.20 b		
62.5	0.00 ± 0.00 b			0.01 ± 0.01 b			0.93 ± 0.45 ab		
31.25	0.10 ± 0.10 b			0.06 ± 0.06 b			1.16 ± 0.35 ab		
EO-2									
Control	1.70 ± 0.11 a	7.33	0.011	1.68 ± 0.26 a	2.99	0.096	2.60 ± 0.17 a	2.79	0.109
15.6	0.60 ± 0.15 b			0.68 ± 0.33 a			1.65 ± 0.29 a		
7.8	1.05 ± 0.25 ab			0.83 ± 0.27 a			2.00 ± 0.30 a		
3.9	1.31 ± 0.13 a			1.25 ± 0.13 a			1.94 ± 0.13 a		
Control	1.71 ± 0.18 a	29.95	<0.001	1.85 ± 0.40 a	21.36	<0.001	2.19 ± 0.12 a	38.97	<0.001
125	0.01 ± 0.01 b			0.02 ± 0.02 b			0.37± 0.15 c		
62.5	0.32 ± 0.22 b			0.02 ± 0.02 b			0.28 ± 0.14 c		
31.25	0.27 ± 0.02 b			0.13 ± 0.08 b			0.93± 0.15 b		
EO-3									
Control	-			1.12 ± 0.21 a	1.265	0.35	-		
1.95	-			0.28 ± 0.28 a			-		
0.97	-			0.53 ± 0.33 a			-		
0.48	-			0.55 ± 0.42 a			-		
Control	1.40 ± 0.41 a	6.166	0.018	1.52 ± 0.22 a	10.06	0.004	-		
15.6	0.08 ± 0.08 b			0.03 ± 0.03 b			-		
7.8	0.43 ± 0.16 b			0.56 ± 0.28 b			-		
3.9	0.62 ± 0.03 ab			0.21 ± 0.21 b			-		
Control	1.14 ± 0.21 a	29.45	<0.001	1.78 ± 0.19 a	73.35	<0.001	2.45 ± 0.13 a	14.3	0.0014
125	0.00 ± 0.00 b			0.00 ± 0.00 b			1.05 ± 0.18 b		
62.5	0.00 ± 0.00 b			0.02 ± 0.02 b			0.88 ± 0.21 b		
31.25	0.02 ± 0.02 b			0.06 ± 0.06 b			1.89 ± 0.24 ab		
α-bisabolol									
Control	1.57 ± 0.11 a	2.235	0.162	1.55 ± 0.22 a			2.07 ± 0.08 a	21.91	<0.001
15.6	0.66 ± 0.29 a			0.27 ± 0.27 b			0.80 ± 0.06 c		
7.8	1.01 ± 0.20 a			0.39 ± 0.19 b			1.36 ± 0.02 b		
3.9	0.85 ± 0.37 a			0.90 ± 0.15 ab			1.70 ± 0.05 ab		
Control	1.97 ± 0.22 a	36.49	<0.001	1.63 ± 0.29 a	29.65	<0.001	2.36 ± 0.07 a	70.63	<0.0001
125	0.05 ± 0.03 b			0.03 ± 0.03 b			0.37 ± 0.15 c		
62.5	0.01 ± 0.00 b			0.00 ± 0.00 b			0.45 ± 0.06 c		
31.25	0.52 ± 0.21 b			0.06 ± 0.05 b			0.86 ± 0.13 b		
DEET									
Control	-			-			1.26 ± 0.19 a	0.24	0.87
15.6	-			-			0.98 ± 0.49 a		
7.8	-			-			1.37 ± 0.28 a		
3.9	-			-			1.16 ± 0.29 a		
Control	1.43 ± 0.19 a	16.24	0.001	1.38 ± 0.25 a	8.9	0.006	1.58 ± 0.11 a	9.71	0.005
125	0.08 ± 0.04 c			0.00 ± 0.00 b			0.42 ± 0.25 b		
62.5	0.74 ± 0.18 b			1.22 ± 0.04 a			0.87 ± 0.13 b		
31.25	1.14 ± 0.10 ab			0.79 ± 0.33 ab			0.84 ± 0.04 b		

^†^ Sand removed is measured in grams. Means within a column, in an experiment, not sharing common letters are significantly different at a level of *p* ≤ 0.05, according to Ryan–Einot–Gabriel–Welsch multiple range test.

**Table 3:** Toxicity of *Matricaria chamomilla* essential oils, a selected pure compound, and bifenthrin against workers of imported fire ants at 24 h post-treatment.

E. oil/Compound	n ^†^	Slope ± SE	LC_50_ (95% CI) ^‡^	LC_90_ (95% CI) ^‡^	χ^2^	*df*
RIFA						
EO-1	30	1.42 ± 0.19	93.60 (76.50–113.29)	230.75 (180.47–336.06)	7.44	13
EO-2	30	1.64 ± 0.22	98.11 (81.98–117.46)	213.63 (169.94–304.02)	12.25	13
EO-3	30	1.75 ± 0.24	142.92 (119.80–169.81)	296.88 (239.67–411.08)	11.46	13
α-bisabolol	30	1.27 ± 0.17	159.23 (129.74–196.81)	434.90 (326.84–679.06)	16.84	13
Bifenthrin	40	1.21 ± 0.18	0.03 (0.023 ± 0.04)	0.09 (0.06 ± 0.16)	42	19
BIFA						
EO-1	30	1.29 ± 0.17	188.11 (153.61–234.68)	504.74 (375.77–800.96)	13.02	13
EO-2	30	1.59 ± 0.21	138.40 (115.23–166.04)	309.21 (245.52–438.35)	4.74	13
EO-3	30	2.78 ± 0.47	202.49 (176.09–233.29)	320.83 (271.53–427.61)	4.28	13
α-bisabolol ^§^	30	-	30%	-	-	-
Bifenthrin	40	1.36 ± 0.23	0.032 (0.023 ± 0.044)	0.08 (0.06 ± 0.15)	34	19
HIFA ***						
EO-1 ^§^	-	-	73%	-	-	-
EO-2 ^§^	-	-	20%	-	-	-
EO-3 ^§^	-	-	40%	-	-	-
α-bisabolol ^§^	-	-	80%	-	-	-
Bifenthrin	40	0.86 ± 0.13	0.018 (0.013 ± 0.024)	0.07 (0.05 ± 0.17)	42.4	22

^†^ n is the number of workers used in each treatment. ^‡^ LC_50_ and LC_90_ values are in µg/g and CIs are confidence intervals. ^§^ Highest mortalities caused at the highest screening dose of 250 µg/g. *** The dose–response curves could not be developed because all the samples showed less than 100% mortality at the highest screening dose of 250 µg/g and abruptly dropped to 0 % at the next serial dose at 24 h post-treatment.

## Data Availability

The data presented in this study are available in the article.
